# A novel dedicated distal cap facilitating deep pancreatic duct insertion during pancreatoscopy

**DOI:** 10.1055/a-2986-1231

**Published:** 2026-10-30

**Authors:** Ryosuke Hamamura, Yuki Tanisaka, Shomei Ryozawa, Masafumi Mizuide, Akashi Fujita, Suguru Ito, Ryuichi Watanabe

**Affiliations:** 1 Department of Gastroenterology 183786 Saitama Medical University International Medical CenterHidakaSaitama PrefectureJapan; 2 GastroenterologySaitama Medical University International Medical CenterHidakaSaitamaJapan; 3 Gastroenterology 183786 Saitama Medical University International Medical CenterHidakaSaitama PrefectureJapan

## A case description



**Video 1** Successful deep pancreatic duct insertion during pancreatoscopy using a novel dedicated distal cap. URI: .


Pancreatoscopy enables direct visualization of the pancreatic duct and is useful for assessing the extent of intraductal lesions and determining the appropriate surgical resection margin in patients with intraductal papillary mucinous neoplasm (IPMN). 1​ ​2​ However, deep insertion of a pancreatoscope can be technically challenging because of the tortuous and angulated anatomy of the pancreatic duct. We report a case in which a novel dedicated distal cap facilitated deep pancreatoscope insertion.

A 65-year-old man was referred to our institution for further evaluation of main pancreatic duct (MPD) dilatation and a pancreatic cystic lesion ( Fig. 1 ). Endoscopic ultrasonography revealed MPD dilatation to 11-mm and a 20-mm multicystic lesion in the pancreatic tail, suggestive of IPMN ( Fig. 2 ). Because the MPD diameter exceeded 10 mm, which is considered a high-risk stigmata, surgical resection was planned. 3​ Endoscopic retrograde cholangiopancreatography was performed to assess lesion extent and determine the appropriate surgical resection margin ( Video 1 ).

**Fig. 1:**
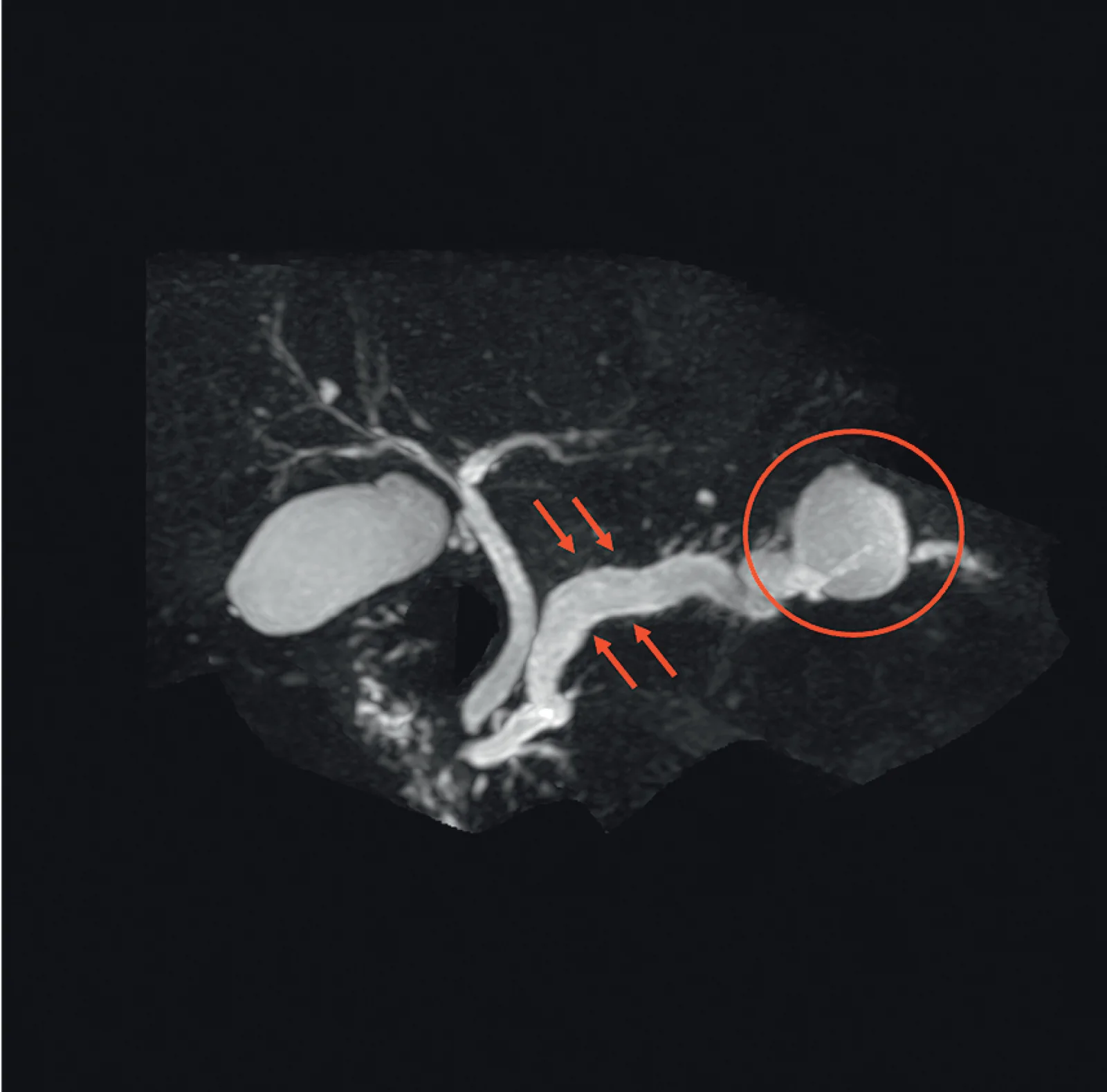
Magnetic resonance cholangiopancreatography revealing the dilated MPD (red arrow) and the cystic lesion (red circle). MPD, main pancreatic duct.

**Fig. 2:**
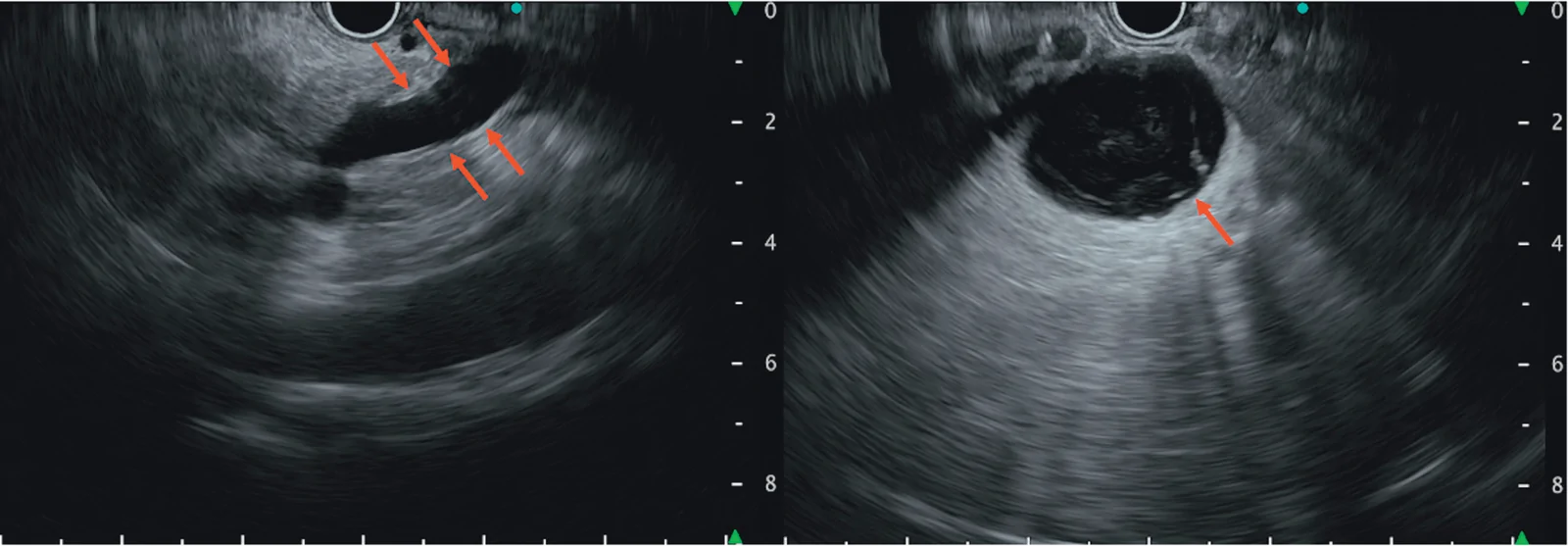
Endoscopic ultrasonography revealing the dilation of the MPD exceeding 10 mm and the multicystic lesion in the pancreatic tail (red arrow). MPD, main pancreatic duct.

Pancreatography revealed a dilated MPD with multiple filling defects caused by abundant mucin and communication between the MPD and the cystic lesion in the pancreatic tail ( Fig. 3 ). Pancreatoscopy was performed using a 9-Fr cholangioscope 4​ ​5​ (eyeMAX; Micro-Tech, China) without a guidewire to preserve scope flexibility because of the tortuous and angulated MPD. A dedicated distal cap (eyeMAX Disposable Distal Cap; Micro-Tech, China; Fig. 4 ) was attached to the tip of the pancreatoscope. The cap facilitated the identification of the distal MPD direction and enabled safe advancement of the pancreatoscope to the pancreatic tail. Pancreatoscopy revealed no findings suggestive of malignancy, such as papillary projections ( Fig. 5 ); therefore, distal pancreatectomy was selected.

**Fig. 3:**
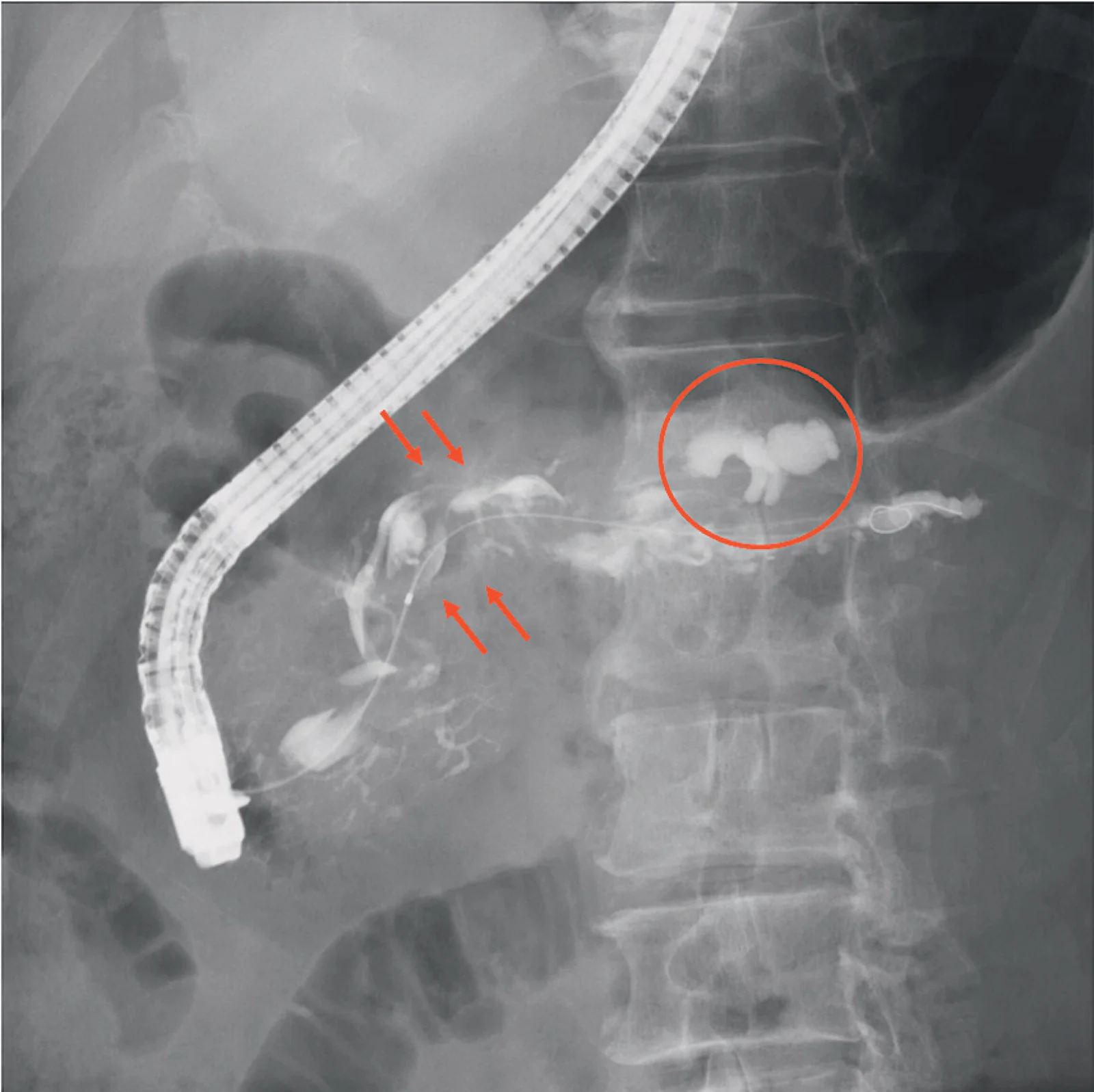
Pancreatography revealing multiple filling defects (red arrow) caused by abundant mucin and communication between the MPD and the cystic lesion (red circle) in the pancreatic tail. MPD, main pancreatic duct.

**Fig. 4:**
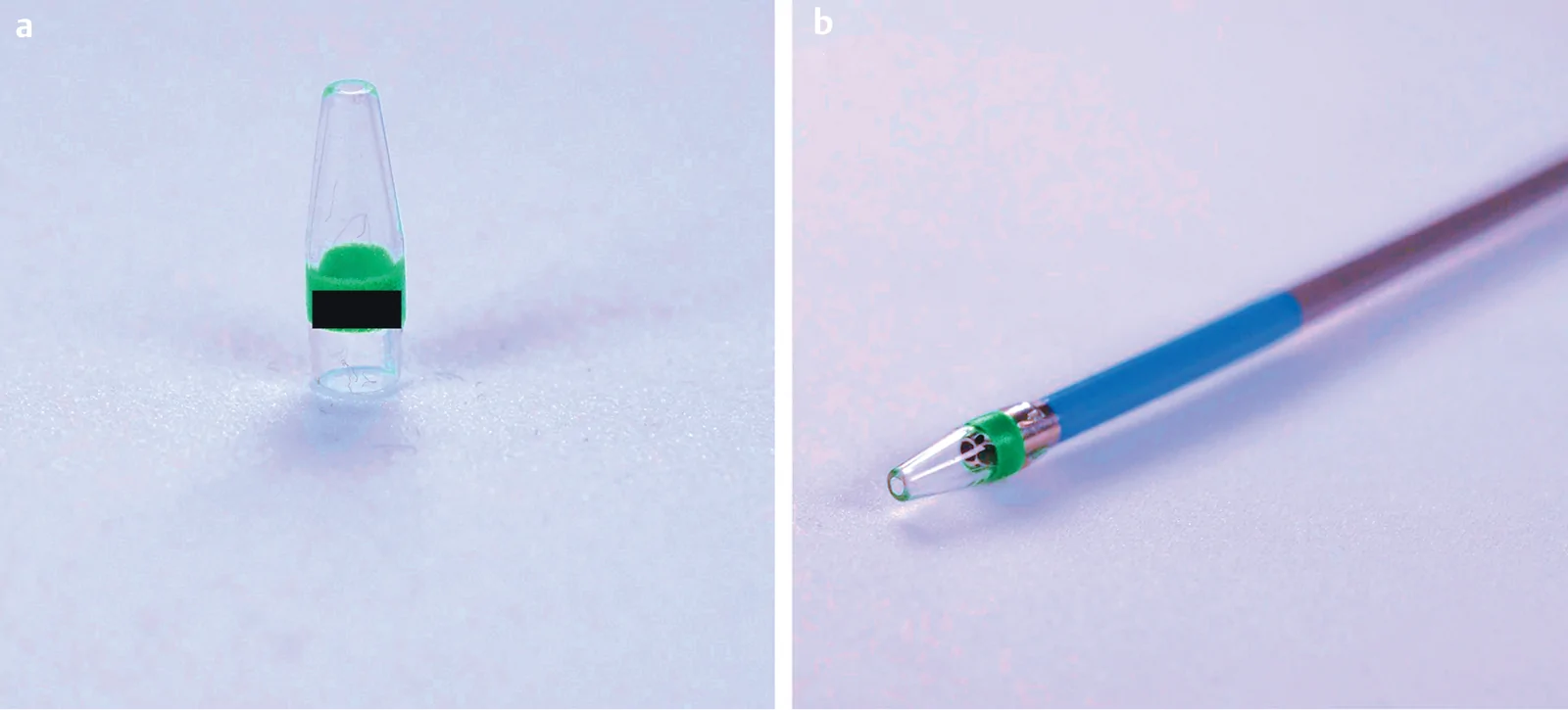
A novel dedicated distal cap (eyeMAX Disposable Distal Cap; Micro-Tech, China) with a tapered tip.

**Fig. 5:**
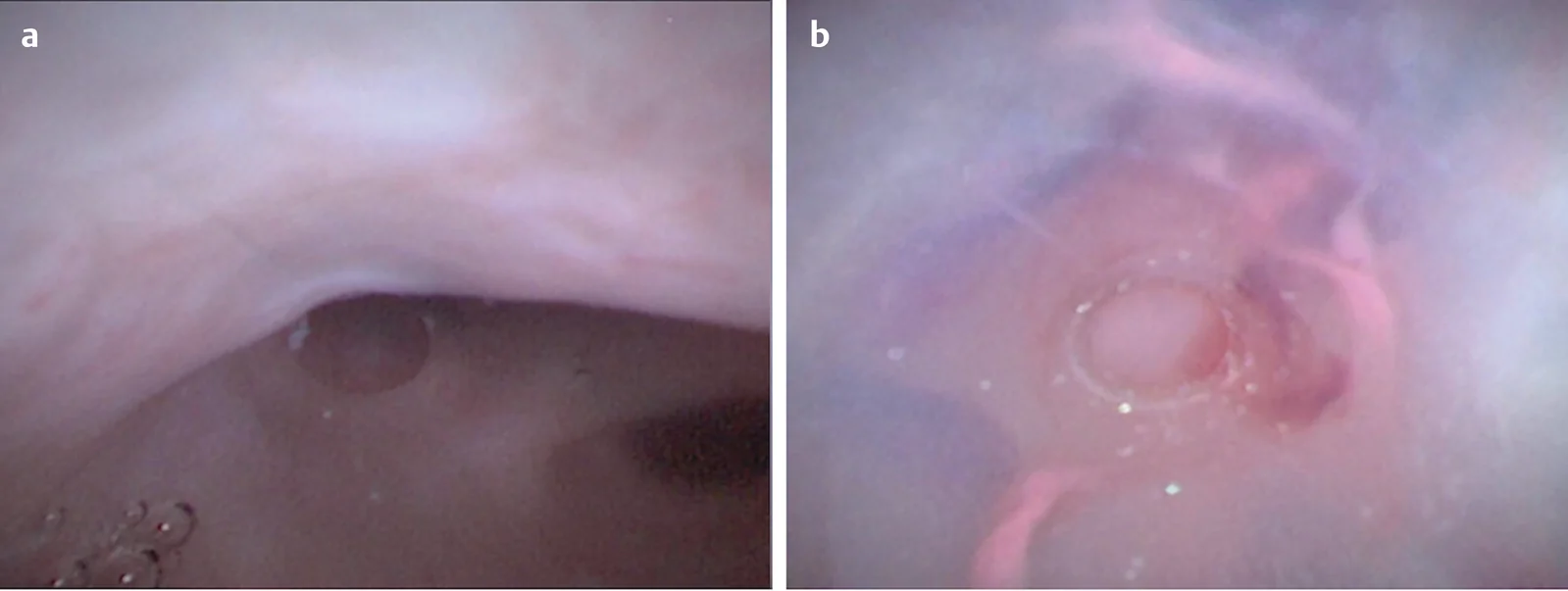
Pancreatoscopic findings. ( **a** ) The dilated MPD is filled with abundant mucin, with no findings suggestive of malignancy. ( **b** ) A pancreatoscopic view inside the pancreatic cyst shows no findings suggestive of malignancy. MPD, main pancreatic duct.

In this case, the dedicated distal cap facilitated deep insertion of the pancreatoscope into the pancreatic duct. This novel accessory may improve the technical feasibility and diagnostic utility of pancreatoscopy in various cases.

Endoscopy_UCTN_Code_TTT_1AR_2AB h

## Declaration of GenAI Use

ChatGPT (OpenAI) was used solely to improve the English language and grammar of the manuscript. It was not used to generate scientific content, analyze data, or draw conclusions. The authors reviewed and approved the final manuscript and take full responsibility for its content.
